# Perceived discrimination, schooling arrangements and psychological adjustments of rural-to-urban migrant children in Beijing, China

**DOI:** 10.1080/21642850.2014.919865

**Published:** 2014-06-17

**Authors:** Lihua Chen, Shaobing Su, Xiaoming Li, Cheuk Chi Tam, Danhua Lin

**Affiliations:** ^a^ Institute of Developmental Psychology, Beijing Normal University, Beijing, People's Republic of China; ^b^ Carman and Ann Adams Department of Pediatrics Prevention Research Center, Wayne State University School of Medicine, Detroit, MI, USA

**Keywords:** rural-to-urban migrant children, psychological adjustments, perceived discrimination, schooling arrangements

## Abstract

*Objectives*: The global literature has revealed potential negative impacts of migration and discrimination on individual's psychological adjustments. However, the psychological adjustments among internal migrant children in developing countries are rarely assessed. This study simultaneously examines perceived discrimination and schooling arrangements in relation to psychological adjustments among rural-to-urban migrant children in China. *Methods*: A sample of 657 migrant children was recruited in Beijing, China. Cross-sectional associations of self-reported perceived discrimination and schooling arrangements (i.e. public school and migrant children school (MCS)) with psychological adjustment outcomes (i.e. social anxiety, depression and loneliness) were examined by general linear model. *Results*: (1) Compared with migrant children in public school, migrant children in MCS had lower family incomes, and their parents had received less education. (2) Migrant children in MCS reported higher levels of social anxiety, depression and loneliness than did their counterparts. Children who reported high level of perceived discrimination also reported the highest level of social anxiety, depression and loneliness. (3) Perceived discrimination had main effects on social anxiety and depression after controlling for the covariates. A significant interaction between perceived discrimination and schooling arrangements on loneliness was found. Specifically, the migrant children in MCS reported higher loneliness scores than did migrant children in public school only at low level of perceived discrimination; however, schooling arrangements was unrelated to loneliness at medium and high levels of discrimination. *Conclusions*: These results indicate that migration-related perceived discrimination is negatively associated with migrant children's psychological adjustments. These findings suggest that effective interventions should be developed to improve migrant children's capacities to cope with migration-related discrimination and improve their psychological adjustments, especially in terms of loneliness.

## 1. Introduction

A substantial global literature suggests that migration, which has been identified as a complex and stressful process, is associated with a broad range of psychological adjustment problems among migrant children (Dogra, Karim, & Ronzoni, 2011), such as depression and anxiety (Diler, Avci, & Seydaoglu, 2003; Oppedal & Røysamb, 2004). Migrant children may also exhibit low self-esteem (Diler et al., 2003), high levels of withdrawal and delinquent behaviors (Janssen et al., 2004). Despite the abundance of existing literature exploring the relationship between international migration and psychological adjustments in the USA and other Western countries, little is known regarding the impact of internal migration on migrant children in developing countries such as China, where internal migration is particularly common.

In China, widespread economic reform and rapid urbanization have caused increasing numbers of rural residents and their children to migrate to urban areas for employment, improved education and better living conditions. Based on the Sixth National Population Census of China in 2010, the number of migrant children under the age of 18 was 35.81 million, which represents a 41.37% increase compared with the national survey in 2005 (Research group of the All-China Women's Federation, 2013). Among these children, 80.35% (28.77 million) are rural-to-urban migrant children. According to the sixth Beijing census data in 2010, Beijing had a population of 19.61 million, and 0.64 million (3.26%) migrant children lived in Beijing without a permanent urban household registration (i.e. “hukou”) (Bureau of Statistics of Beijing, 2013).

Discrimination is demonstrated to be one of the stressors, which migrant children are facing in urban areas (Fang, Fan, & Liu, 2008; Lin, Fang, Liu, & Lan, 2009). Migrant populations are often discriminated against in urban areas because they are believed to have some undesirable attributes or characteristics (e.g. low socioeconomic status (SES), accent, undesirable appearance and behavior) that mark them as different and cause them to be devalued, rejected and excluded (Li et al., 2007, 2010; Link & Phelan, 2001; Major & O'Brien, 2005). By limiting access to important life domains, discrimination has direct and enormous negative effect on adolescents' psychological adjustments and physical health that include depression, anxiety, lower self-esteem, hypertension and coronary heart disease (Greene, Way, & Pahl, 2006; Link & Phelan, 2001; Major & O'Brien, 2005; Virta, Sam, & Westin, 2004). Additionally, researchers have found that perceived discrimination is negatively and significantly related to both psychological and sociocultural adaptations among immigrant youth (Berry, Phinney, Sam, & Vedder, 2006). Although existing studies generally suggest that perceived discrimination is inversely associated with psychological adjustments, limited data exist regarding the relationship between discrimination and psychological adjustments in rural-to-urban migrant populations, especially in migrant children. Consistent with evidence gathered from immigrant youth and rural-to-urban migrants (Berry et al., 2006; Lin et al., 2011; Wang, Li, Stanton, & Fang, 2010), a few studies conducted among rural-to-urban migrant children in China have found that perceived discrimination directly affected children's psychological adjustments by causing depression, social anxiety and loneliness (Fang et al., 2008; Lin et al., 2009).

Due to the restrictions imposed by the household registration system of the Chinese government, migrant children encounter unequal schooling opportunities in urban areas. Currently, there are two main types of schooling arrangements for school-age (i.e. 6–14 years of age) migrant children to complete their compulsory education in urban areas (i.e. they can transiently attend public school or they can attend migrant children school (MCS) specifically for migrant children). Compared with public school, MCS is more likely to be unlicensed and has inferior teaching conditions and poorly qualified teachers (Han, 2001a, 2001b; Li et al., 2010). Additionally, the relocation of MCS occurs frequently (Su, Tam, Chen, & Lin, 2013), reflecting an unstable educational environment for migrant children. The limited existing literature on this subject reveals that schooling arrangements is associated with migrant children's psychological adjustments (Lin et al., 2009; Yuan, Fang, Liu, & Li, 2009; Zhou, 2006); however, the limited existing studies about the differences between children in MCS and those in public school have yielded mixed results. For example, some researchers have suggested that migrant children in public school are generally more likely to report higher level of loneliness than those in MCS (Zeng, 2008; Zhou, 2006). In contrast, others have suggested that migrant children in MCS are more likely to report higher levels of loneliness and depression compared with their counterparts in public school (Lin et al., 2009; Yuan et al., 2009). Thus, it is important to explore the association of schooling arrangements and psychological adjustments among rural-to-urban migrant children in China.

This study was designed to explore psychological adjustments of migrant children in Beijing, China, and answer the following research questions: (1) What are the demographic characteristics of these two groups of children (i.e. migrant children in public school and migrant children in MCS)? (2) Do children's psychological adjustment outcomes (i.e. social anxiety, depression and loneliness) differ by children's schooling arrangements and levels of perceived discrimination? (3) Does perceived discrimination significantly interact with schooling arrangements on migrant children's psychological adjustments?

## 2. Method

### 2.1. Study population and procedures

The sample of this study was recruited in the baseline survey for a longitudinal study that has been conducted since 2011 in Beijing, China. Beijing is the capital city of China and covers approximately 16,410 km^2^. The Beijing municipal government has jurisdiction over two central urban districts, four near-suburban districts, eight outer-suburban districts and two counties. The sample was recruited from migrant children in the fourth, fifth and sixth grades of three elementary schools (two public schools and one MCS) in the Chaoyang and Daxing districts of Beijing, China. To recruit migrant children from the elementary schools, we worked with the school principals to generate lists of eligible migrant children. The eligibility criteria included the following: (1) no “hukou” in Beijing and (2) living with parents who had migrated to Beijing for employment more than three months previously. After acquiring permission from the school principals to conduct the survey in their schools, trained interviewers (15 graduate students majoring in psychology from Beijing Normal University) invited the children to participate in the survey. Eligible children who agreed to participate and provided informed consent were asked to complete a battery of self-administered questionnaires. All participants completed the survey in their classrooms.

The ﬁnal sample consisted of 657 migrant children, including 300 (45.7%) migrant children in MCS and 357 (54.3%) migrant children in public school. Of the 657 participants, 209 (31.8%) were fourth graders, 213 (32.4%) were fifth graders and 235 (35.8%) were sixth graders.

### 2.2. Measures

#### 2.2.1. Demographic characteristics

All participants were asked to provide several demographic characteristics that included gender, parents' educational levels (i.e. elementary school or lower, middle school and high school or higher) and gross monthly family income (in Chinese Yuans (6.3 Yuan = 1 US dollar at the time of survey), i.e. less than 2000 Yuan, 2000–6000 Yuan and higher than 6000 Yuan). To obtain estimates of the family SES, a composite SES score was created by indexing children for whom both parents’ education that extended past elementary school and monthly family incomes of 2000 Yuan or higher. The composite SES score ranged from 0 to 3, and higher scores indicated better family SES.

#### 2.2.2. Perceived discrimination

The Perceived Discrimination Scale for Migrant Children was developed based on existing measures of perceived discrimination in the literature and interview results from migrant children, their parents and teachers (Liu & Shen, 2010). The scale has shown good internal reliability and validity in previous studies (Liu, 2013; Liu, Zhao, & Shen, 2013). The scale consists of 17 items that assess migrant children's perceived discrimination in their daily lives in Beijing. The items are based on a 4-point scale that ranges from 1 (not at all true) to 4 (always true), and higher scores indicate higher levels of perceived discrimination. The Cronbach's *α* for this scale was 0.86 in this sample.

#### 2.2.3. Social anxiety

Social anxiety was assessed with the 10-item Social Anxiety Scale for Children (La Greca, Dandes, Wick, Shaw, & Stone, 1988). Children were asked to self-report how often situations related to social anxiety happened in their daily lives (1 = never, 2 = occasionally, 3 = sometimes and 4 = always). Composite scores of 10 items were obtained, and higher scores indicated higher levels of social anxiety. The Cronbach's *α* for this scale was 0.87 in this sample.

#### 2.2.4. Depression

The self-reported 20-item Center for Epidemiologic Studies Depression Scale for Children (CES-DC) (Fendrich, Weissman, & Warner, 1990) was used to measure depression in this study. The children were asked to self-report how frequently situations and feelings related to depressive symptoms had happened in the previous week (1 = never, 2 = occasionally, 3 = sometimes and 4 = always). Composite scores were obtained, and higher scores indicated higher levels of depression. The Cronbach's *α* for this scale was 0.84 in this sample.

#### 2.2.5. Loneliness

The Loneliness Scale for Children (Asher, Hymel, & Renshaw, 1984) was used to assess the children's perceived loneliness and social dissatisfaction. The scale consists of 16 items that focus on the children's feelings of loneliness (e.g. “I'm lonely”) and subjective estimations of peer status (e.g. “I have lots of friends”). Responses to the items are given on a 5-point Likert scale (1 = always true, 2 = true most of the time, 3 = true sometimes, 4 = hardly ever true and 5 = not true at all), and higher scores indicate higher levels of loneliness. The Cronbach's *α* for this scale was 0.91 in this sample.

### 2.3. Statistical analyses

First, chi-square test (for categorical variables) or *t-*test (for continuous variable) was employed to examine differences in key demographic characteristics between migrant children in MCS and those in public school. Second, analysis of variance or *t-*test was employed to examine group differences in terms of social anxiety, depression and loneliness. A categorical score was created by dividing children into three groups (i.e. approximately the bottom 25%, middle 50% and top 25%) based on their scores on the discrimination scale and used as a between-subjects factor. Finally, a general linear model (GLM) was employed to simultaneously assess the effects of perceived discrimination and schooling arrangements on the migrant children's psychological adjustments after controlling for gender and SES. All statistical analyses were performed using SPSS for Windows 18.0.

## 3. Results

### 3.1. Sample characteristics

 Table 1 presents descriptive statistics for the rural-to-urban migrant children in MCS (*N *= 300) and public school (*N *= 357) in this sample. The entire sample consisted of 260 females (40.4%) and 383 males (59.6%). The mean SES score for the sample was 2.31 (SD = 0.90). Migrant children in MCS differed from those in public school in all characteristics except gender. Compared with migrant children in public school, more migrant children in MCS reported that their fathers (22.2% vs. 6.9%) and mothers (39.1% vs. 12.2%) had lower levels of educational attainment (i.e. no more than elementary school education). Additionally, migrant children in MCS were about twice as likely as those in public school to report lower levels of family income (42.4% vs. 19.5%). The SES of migrant children in MCS were significantly lower than those of migrant children in public school (1.96 vs. 2.60, *p *< .001).

**Table 1.** Demographic characteristics of the sample.

	Overall	Migrant children in MCS	Migrant children in public school	*χ*^2^	*p*
*n* (%)	657 (100%)	300 (45.7%)	357 (54.3%)	4.95	.026
Gender				0.44	.506
Female	260 (40.4%)	123 (41.8%)	137 (39.3%)		
Male	383 (59.6%)	171 (58.2%)	212 (60.7%)		
Father's educational level				60.43	<.001
Elementary school or lower	87 (13.7%)	63 (22.2%)	24 (6.9%)		
Middle school	276 (43.5%)	143 (50.4%)	133 (38.0%)		
High school or higher	271 (42.7%)	78 (27.5%)	193 (55.1%)		
Mother's educational level				89.37	<.001
Elementary school or lower	149 (23.9%)	106 (39.1%)	43 (12.2%)		
Middle school	239 (38.4%)	112 (41.3%)	127 (36.1%)		
High school or higher	235 (37.7%)	53 (19.6%)	182 (51.7%)		
Family income				51.36	<.001
Lower than 2000 Yuan	185 (29.6%)	117 (42.4%)	68 (19.5%)		
2000–6000 Yuan	250 (40.1%)	109 (39.5%)	141 (40.5%)		
Higher than 6000 Yuan	189 (30.3%)	50 (18.1%)	139 (39.9%)		
SES, *M* (SD)^a^	2.31 (0.90)	1.96 (1.01)	2.60 (0.66)	9.49	<.001

^a^Student's *t*-test.

*M*, mean; SD, standard deviation.

Note: Sample sizes differed slightly across analyses owing to missing self-report of individual characteristics for some participants.

### 3.2. Relationships of psychological adjustment, perceived discrimination and schooling arrangements

Table 2 presents the group differences in social anxiety, depression and loneliness by perceived discrimination and schooling arrangements. Perceived discrimination was found to be significantly associated with all psychological adjustment outcomes. *Post hoc* comparisons revealed that children with high level of perceived discrimination exhibited the highest social anxiety, depression and loneliness scores among all the participants. Additionally, children with medium level of perceived discrimination reported higher social anxiety, depression and loneliness scores than did children with low level of perceived discrimination. Additionally, compared with migrant children in public school, migrant children in MCS exhibited higher scores for psychological adjustment problems, including social anxiety, depression and loneliness.

**Table 2.** Group differences in terms of social anxiety, depression and loneliness.

	Perceived discrimination^a^				Schooling arrangements	
	Low (1)	Medium (2)	High (3)	*Post hoc* comparison	MCS	Public school
Social anxiety, *M* (SD)	1.34 (0.44)	1.57 (0.52)	2.06 (0.72)***	(1,2)(1,3)(2,3)	1.77 (.61)	1.52 (.61)***
Depression, *M* (SD)	1.59 (0.35)	1.72 (0.37)	2.20 (0.53)***	(1,2)(1,3)(2,3)	1.93 (.45)	1.71 (.47)***
Loneliness, *M* (SD)	1.71 (0.77)	1.91 (0.73)	2.54 (0.80)***	(1,2)(1,3)(2,3)	2.24 (.72)	1.84 (.86)***

^a^Perceived discrimination: low perceived discrimination, medium perceived discrimination and high perceived discrimination.

*p < .05.

**p < .01.

***p < .001.

### 3.3. Multivariate analysis

The GLM analysis of social anxiety, depression and loneliness (Table 3) revealed a significant difference between children with different levels of perceived discrimination in the multivariate test (*p *< .001) after controlling for gender and family SES. A significant schooling arrangements difference was found in the multivariate test (*p *< .05) after controlling for the covariates. Additionally, a significant interaction between the levels of perceived discrimination and schooling arrangements was found in the multivariate test (*p *< .01). Moreover, both the gender and SES were significant covariates in the multivariate test (*ps *< .05).

**Table 3.** GLM analysis of social anxiety, depression and loneliness.

	Main effect		Interaction	Covariates	
	Perceived discrimination	Schooling arrangements	Discrimination × schooling arrangements	Gender	SES
Multivariate test (Wilks' Lambda)	30.18***	3.05*	3.28**	3.22*	3.66*
Social anxiety	59.99***	1.18	0.14	5.20*	3.51
Depression	77.13***	3.53	2.35	0.03	6.38*
Loneliness	38.95***	8.94**	9.05***	0.79	9.73**

*p < .05.

**p < .01.

***p < .001.

**Figure 1.**
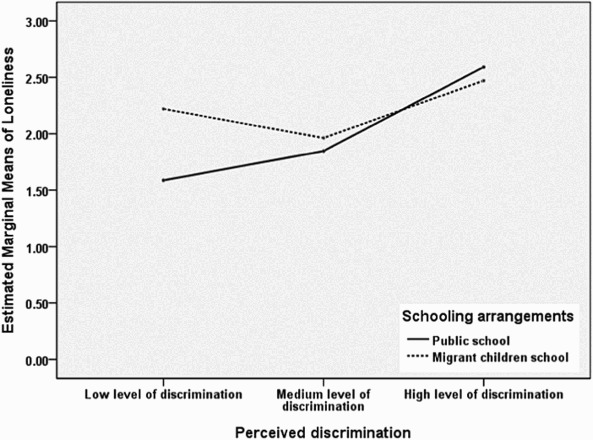
Profile plot of loneliness.

Furthermore, the results showed that perceived discrimination had main effects on social anxiety (*p *< .001) and depression (*p *< .001) after controlling for gender and SES. Pairwise comparisons revealed that children with high level of perceived discrimination exhibited the highest social anxiety and depression among all the participants (*ps *< .01). The interaction between perceived discrimination and schooling arrangements was significant only for loneliness (*p *< .001). Further examination of estimated marginal means (Figure 1) revealed significant difference in loneliness between children in MCS and those in public school only at low level of discrimination (*p *< .001), specifically, migrant children in MCS reported higher level of loneliness than their counterparts in public school. However, schooling arrangements was unrelated to loneliness at medium and high levels of perceived discrimination (*ps *> .05). Regarding the covariates, gender was associated with social anxiety (*p *< 0.05), and SES was associated with both depression (*p *< .05) and loneliness (*p *< .01).

## 4. Discussion

One of the contributions of this study is the comparison in the characteristics of migrant children in MCS and those in public school. First, the results showed that, compared with parents of migrant children in public school, parents of migrant children in MCS had lower level of educational attainment and lower family incomes. These findings are consistent with those of another study that was conducted in China (Yuan et al., 2009), which indicate that migrant children in public school have better family SES. Second, the ﬁndings revealed that migrant children in MCS experienced higher levels of social anxiety, depression and loneliness compared with those in public school, which is consistent with most previous studies (Lin et al., 2009; Yuan et al., 2009), indicating that migrant children's psychological adjustment status vary across different schooling arrangements. As suggested by these findings, migrant children in MCS are currently at a relative disadvantage in terms of psychological adjustment outcomes, requiring more attention and support from communities, schools and families to improve their mental health.

Our main findings included a significant main effect of perceived discrimination on migrant children's psychological adjustment outcomes, which is consistent with the previous studies on the negative effects of discrimination on individuals' psychological adjustments (Fang et al., 2008; Lin et al., 2009, 2011; Link & Phelan 2001; Liu, 2013; Liu, & Shen, 2010). It should be noted that perceived discrimination contributes to both children's social anxiety and depression independently of their schooling arrangements and other key demographic factors. These results provide strong evidence that perceived discrimination in daily life is negatively associated with psychological adjustments, which implies that the impact of migration-related perceived discrimination on migrant children's psychological adjustments could be enormous and stable. Our findings also highlight the importance of exploring the factors that contribute to discrimination and the potential mechanisms by which discrimination affects migrant children's psychological adjustments in further studies.

This study also found a significant interaction effect between perceived discrimination and schooling arrangements on loneliness; specifically, migrant children in MCS reported higher level of loneliness than those in public school only at low level of discrimination. As suggested by these findings, for migrant children who suffered low level of discrimination, there might be some other potential stressors associating with their loneliness, including children's frequency of transferring schools in urban areas (Hu, Fang, Lin, & Liu, 2009). Because the relocation and shutdown of MCS occurs frequently, children in MCS transfer schools more frequently than those in public school, which might lead to unstable relationships with peer and teachers for children in MCS, and result in their higher level of loneliness (Hu et al., 2009; Su et al., 2013). However, it should be noted that there was no significant difference in loneliness between migrant children in MCS and those in public school at medium and high levels of discrimination, which showed a strong correlation between high level of perceived discrimination and high level of loneliness irrespective of children's schooling arrangements, demonstrating the considerable detrimental effect of discrimination on children's loneliness. As suggested by these findings, special attention should be paid to all the migrant children who suffered high level of perceived discrimination to improve their psychological adjustments.

Several potential limitations of this study should be noted. First, despite our efforts to ensure the representativeness of the samples, our samples were convenience samples of rural-to-urban migrant children in Beijing, which limits our ability to generalize our findings to migrant children in other areas of China. Second, the sample was recruited from schools, which limits our ability to examine the impact of perceived discrimination on the psychological adjustments of children who are not attending school. Finally, relying on children to report information about their parents may have led to inaccuracies and unreliabilities in the results. The use of tools that directly assess the parents in further research will be of great value.

Regardless of these limitations, this study also has important implications for child welfare policy and practice. First, future preventative interventions should seek to increase public attention to the psychological adjustments of migrant children and promote the psychological adjustments of migrant children, especially for migrant children in MCS. Second, our ﬁndings underscore the need to reduce public discrimination against rural-to-urban migrant children. Future work should seek to improve public attitudes toward rural-to-urban migrant children by acknowledging their parents' contributions to the urban socioeconomic development of China and to generate policies that eliminate or reduce discrimination and prejudicial attitudes toward migrant populations. Most importantly, it is essential to create non-discriminatory atmospheres in schools to ensure the positive development of migrant children. Finally, effective school-appropriate intervention programs should be developed to improve migrant children's abilities to cope with migration-related discrimination.

## Funding

This research was supported by Beijing Excellent Talents training Foundation [2011D0090 12000003].

## Appendix 1. The items of the Perceived Discrimination Scale for Migrant Children (Liu & Shen, 2010)

1. I need to pay a lot of money for attending a public school in Beijing.
2. I am not eligible to take the high school or College Entrance Examination in Beijing, I have to go back hometown to take the exam.
3. Some public schools in Beijing are unwilling to accept me.
4. Children in Beijing are unwilling to study together with me.
5. Some children in Beijing do not see me as intelligently qualified.
6. Some children in the community or school in Beijing are not friendly to me.
7. Some children in the community or school in Beijing are not willing to hang out with me and even avoid me.
8. The tones in which people in Beijing talk to me annoy me.
9. The ways people in Beijing look at me make me feel uncomfortable.
10. When asked for help, people in Beijing are impatient with me.
11. Some children in the community or school in Beijing view me as a dishonest person.
12. Some children in the community or school in Beijing laugh at me for my non-standard mandarin.
13. Some children in the community or school in Beijing laugh at me for being poorly dressed.
14. Children in the community or school in Beijing laugh at me for being unhygienic.
15. I have been nicknamed by some children in the community or school in Beijing.
16. I have been hit or cursed by some children in the community or school in Beijing.
17. When I go out and play or go shopping, I will be bullied by children in Beijing.
